# In-silico EEG biomarkers of reduced inhibition in human cortical microcircuits in depression

**DOI:** 10.1371/journal.pcbi.1010986

**Published:** 2023-04-10

**Authors:** Frank Mazza, Alexandre Guet-McCreight, Taufik A. Valiante, John D. Griffiths, Etay Hay

**Affiliations:** 1 Krembil Centre for Neuroinformatics, Centre for Addiction and Mental Health, Toronto, Canada; 2 Department of Physiology, University of Toronto, Canada; 3 Krembil Brain Institute, University Healthy Network, Toronto, Canada; 4 Department of Electrical and Computer Engineering, University of Toronto, Toronto, Canada; 5 Institute of Biomaterials and Biomedical Engineering, University of Toronto, Toronto, Canada; 6 Department of Surgery, University of Toronto, Toronto, Canada; 7 Center for Advancing Neurotechnological Innovation to Application, Toronto, Canada; 8 Max Planck-University of Toronto Center for Neural Science and Technology, Toronto, Canada; 9 Institute of Medical Sciences, University of Toronto, Toronto, Canada; 10 Department of Psychiatry, University of Toronto, Toronto, Canada; University of Oxford, UNITED KINGDOM

## Abstract

Reduced cortical inhibition by somatostatin-expressing (SST) interneurons has been strongly associated with treatment-resistant depression. However, due to technical limitations it is impossible to establish experimentally in humans whether the effects of reduced SST interneuron inhibition on microcircuit activity have signatures detectable in clinically-relevant brain signals such as electroencephalography (EEG). To overcome these limitations, we simulated resting-state activity and EEG using detailed models of human cortical microcircuits with normal (healthy) or reduced SST interneuron inhibition (depression), and found that depression microcircuits exhibited increased theta, alpha and low beta power (4–16 Hz). The changes in depression involved a combination of an aperiodic broadband and periodic theta components. We then demonstrated the specificity of the EEG signatures of reduced SST interneuron inhibition by showing they were distinct from those corresponding to reduced parvalbumin-expressing (PV) interneuron inhibition. Our study thus links SST interneuron inhibition level to distinct features in EEG simulated from detailed human microcircuits, which can serve to better identify mechanistic subtypes of depression using EEG, and non-invasively monitor modulation of cortical inhibition.

## Author summary

Reduced somatostatin-expressing interneuron (SST) inhibition has been implicated in depression. However, it is impossible to establish experimentally in humans how these inhibitory changes are reflected in clinically relevant brain signals (electroencephalography, EEG). We performed detailed simulations of human neuronal network activity and EEG signals in health and depression. We found that reduced SST inhibition led to significant changes in EEG, which accounts for changes seen in depression. Our study thus provides biomarkers that link inhibition level of a key interneuron type to measurable signatures in EEG. These biomarkers can be used to identify subtypes of depression and non-invasively monitor cortical inhibition modulation.

## Introduction

Major depressive disorder (depression) is a leading cause of disability worldwide [1] and involves varying mechanisms and symptoms [2,3]. Consequently, a significant proportion of patients remain resistant to antidepressants [4] and second-line treatments [5]. Electro-encephalography (EEG) offers a non-invasive and cost-effective method for brain signal-based biomarkers to improve diagnosis, monitoring and treatment of depression subtypes [6,7]. However, the multi-scale mechanisms of depression and their link to clinically-relevant brain signals remain poorly understood.

There is growing evidence that reduced cortical inhibition plays a mechanistic role in depression [8] and treatment-resistant depression [9–11], especially inhibition mediated by somatostatin-expressing (SST) interneurons [11–15]. Recent studies showed a marked reduction in SST expression by SST interneurons in post-mortem tissue of depression patients, across all layers of the prefrontal cortex (PFC) and anterior cingulate cortex–key regions implicated in depression [16–18]. Reduced SST expression by SST interneurons would mechanistically present as reduced synaptic and tonic inhibition by SST interneurons since SST and gamma-aminobutyric-acid (GABA) receptors are colocalized, and SST is co-released with GABA to strengthen inhibitory effects through postsynaptic mechanisms [19]. In rodent models, SST knockout mice show altered GABAergic genes in SST interneurons consistent with reduced GABA function [20]. Relatedly, silencing SST interneuron inhibition led to depression symptoms [15], and novel therapeutic compounds acting via positive allosteric modulation of alpha-5-GABA-A (*α*5-GABA_A_) receptors targeted by SST interneurons resulted in pro-cognitive and anxiolytic effects [15,21]. In contrast, indications of changes in parvalbumin-expressing (PV) interneuron inhibition were inconsistent and less pronounced, signifying a more selective vulnerability for SST interneurons in depression [22,23]. Other disorders such as Alzheimer’s and aging that show changes in SST interneuron inhibition involve other key changes such as cell and synapse loss, therefore the altered inhibition may play a less central or less consistent role in these conditions than in depression [24–26].

The effects of reduced SST inhibition on circuit activity could have signatures detectible in EEG, due to this cell type’s principal role in modulating input to pyramidal (Pyr) neurons in layer 2/3, which is closest to surface EEG electrodes, as well as layer 5/6 Pyr neurons whose dendrites reach the superficial layers. SST interneurons provide synaptic and tonic inhibition onto the apical dendrites of Pyr neurons [27,28], and mediate lateral inhibition in the cortex [29–31], particularly during periods of quiet, resting wakefulness (resting state) [32]. Accordingly, previous studies indicate that reduced SST interneuron inhibition in depression increases baseline activity of Pyr neurons [12,29,33]. While the contribution of SST interneurons to resting-state cortical oscillations remains largely unknown, studies show a role for this cell type in modulating low-frequency oscillations. SST stimulation entrains network activity in the 5–30 Hz range, and SST suppression modulates theta band (4–8 Hz) power [34,35]. SST interneurons have also been suggested to govern network synchrony in slow-wave sleep, which is marked by slow oscillations [36]. Thus, reduced SST interneuron inhibition in depression may affect EEG low-frequency power. Conversely, PV interneurons have been shown to modulate high beta (20–30 Hz) and gamma (30–50 Hz) frequencies, indicating that these two interneuron types likely modulate distinct frequency domains in recorded EEG [37,38]. We recently showed that a 40% reduction in SST interneuron inhibition, estimated from post-mortem tissue studies of depression patients [16], significantly increased baseline Pyr spike rate in simulated human microcircuits [33]. However, it is still unknown if this level of reduction in SST interneuron inhibition would significantly alter baseline oscillatory dynamics detectable in EEG.

Previous studies have linked neuronal spiking to extracellular signals, although mostly using animal models and local field potential (LFP) [39–41]. Human studies have characterized LFP oscillations in cortical slices and showed phase-amplitude coupling between deep and superficial layers [42]. Others have studied the phase preference of Pyr neuron spikes relative to spindle events in rodent intracranial EEG oscillations [43]. However, experimental methods are limited in their ability to characterize the effects of the cellular changes in depression on human brain signals *in vivo*, thus meriting the use of computational models. Previous computational studies have identified inter-laminar mechanisms underlying evoked related potentials during stimulus response using simplified neuron morphologies and connectivity [44,45]. The increased availability of human neuronal electrical and synaptic connectivity data [31,46,47] provides important constraints for detailed models of human cortical microcircuits [33], which can be used to link mechanisms of microcircuit activity in human health and disease to signatures in local circuit-generated EEG signals [48,49].

In this study, we identified EEG biomarkers of microcircuit effects due to reduced SST interneuron inhibition, as estimated from gene expression changes in depression [16,33]. Using biophysically detailed models of human cortical microcircuits, we simulated resting-state activity in health and depression together with local EEG signals. We characterized changes in resting-state EEG spectral power, and in relation to spiking activity in different neuron types, to identify biomarkers of reduced SST interneuron inhibition in depression.

## Results

### Human cortical microcircuit models reproduce resting-state EEG features

**Fig 1:**
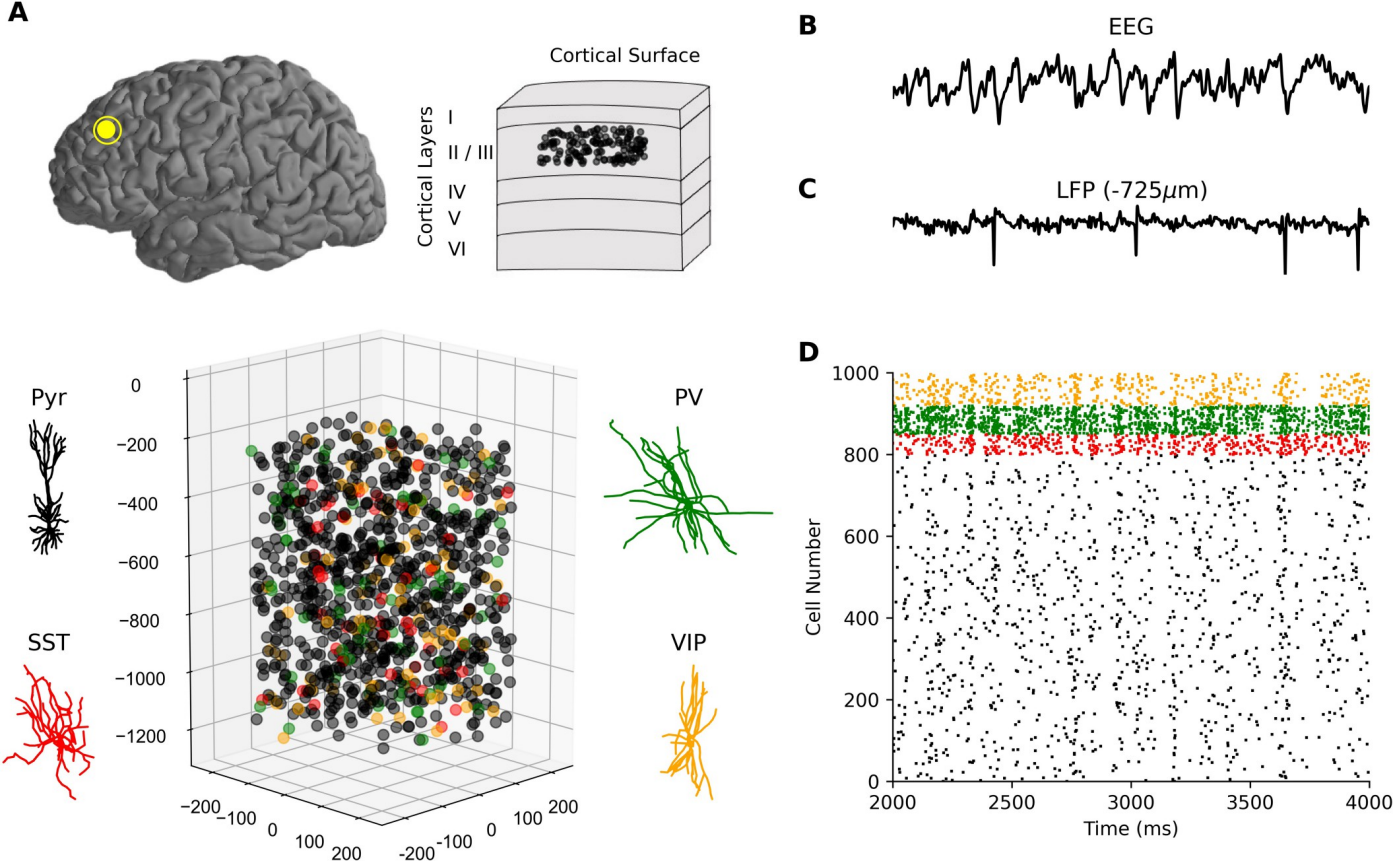
Simulating neuronal spiking and EEG signals from human cortical microcircuits. **(a)** Detailed models of human cortical microcircuits, showing somatic placement of 1000 neurons in a 500x500x950 μm^3^ volume along layer 2/3 (250–1200 μm below pia) and reconstructed morphologies used in the neuron models. **(b–d)** Temporally aligned multi-scale simulated signals: EEG (b) from scalp electrode directly above the microcircuit; LFP signal (c) recorded at the middle of L2/3 (depth of -725 μm); Raster plot of spiking in different neurons in the microcircuit (d), color-coded according to neuron type. Neurons received background excitatory inputs to generate intrinsic circuit activity.

We used our previous detailed models of human cortical L2/3 microcircuits [33] (Fig 1A) as canonical cortical microcircuit models for simulating resting state spiking and EEG signals. The model microcircuits included the four key neuron types: Pyr neurons, SST interneurons, Parvalbumin-expressing interneurons (PV), and Vasoactive intestinal polypeptide-expressing interneurons (VIP). To simulate intrinsic healthy resting-state spiking activity, all neurons received random background excitatory input corresponding to baseline cortical and thalamic drive. The model microcircuits were implemented in a physical volume, enabling simulation of LFP and EEG together with microcircuit spiking (Fig 1A–1D).

**Fig 2:**
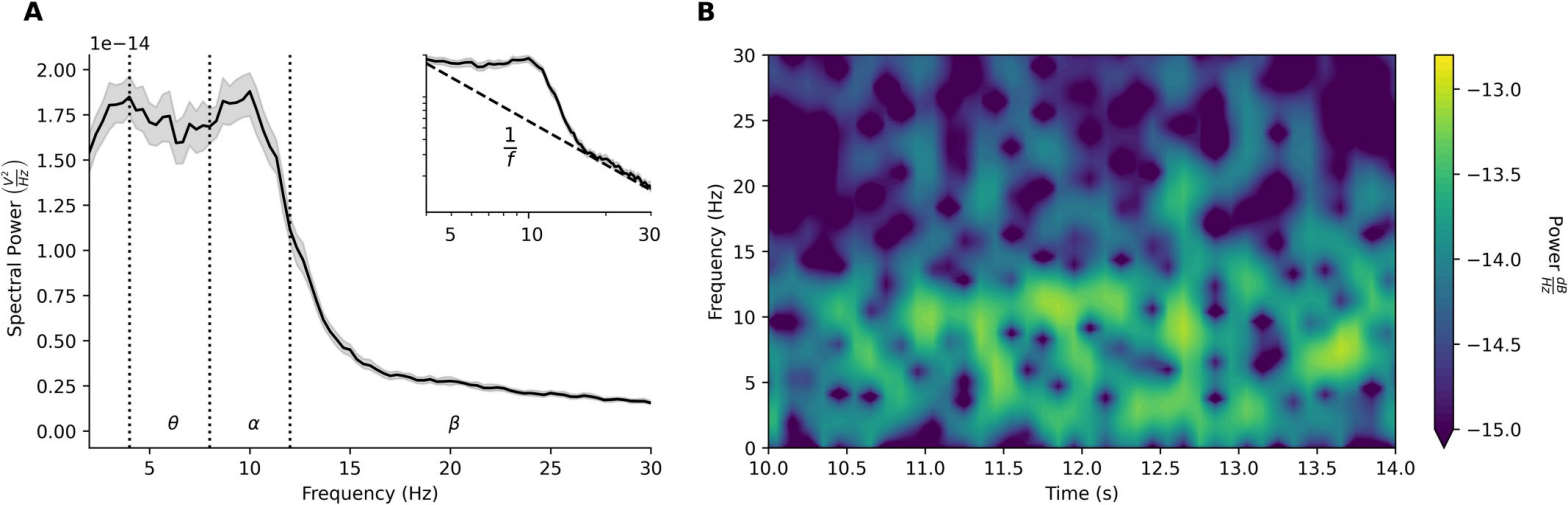
Models of human cortical microcircuits reproduce key features of resting-state EEG. **(a)** Power spectral density plot of circuit simulations (n = 60 randomized microcircuits, bootstrapped mean and 95% confidence intervals), exhibiting peak power in theta and alpha frequency bands. Inset–same power spectral density plot shown on log-log scale, illustrating the 1/f relationship between power and log frequency, inversely linear between 3–30 Hz with slope -1.17 ± 0.09. Frequency bands are delimited by dotted lines. **(b)** Example spectrogram of simulated microcircuit EEG, exhibiting theta and alpha events.

Baseline microcircuit activity was oscillatory, marked by synchronous spiking events (Fig 1D) and corresponding fluctuations in LFP and EEG signals (Fig 1B and 1C). We quantified oscillatory activity in the healthy microcircuits by calculating EEG power spectral density (PSD). The microcircuit EEG exhibited a peak in theta (4–8 Hz) and alpha (8–12 Hz) bands (*n* = 60 randomized microcircuits, Fig 2A) and a 1/f background trend (Fig 2A, inset). We then calculated spectrograms of circuit activity to analyze the evolution of signal strength in the time-frequency domain. The spectrograms showed 41 ± 3 transient theta-alpha events per 28 s simulation, with average duration of 181 ± 12 ms (Fig 2B). The circuit simulations thus reproduced several key temporal and spectral features of resting-state human EEG, including an oscillatory peak power in theta and alpha bands and a background 1/f trend [50–52]. Critically, these oscillatory properties were emergent, and were not explicitly optimized for, which therefore constitutes an important validation of the model and demonstration of its ability to capture key properties of human cortical microcircuit dynamics and associated EEG signals.

### EEG biomarkers of reduced SST in depression microcircuits

**Fig 3:**
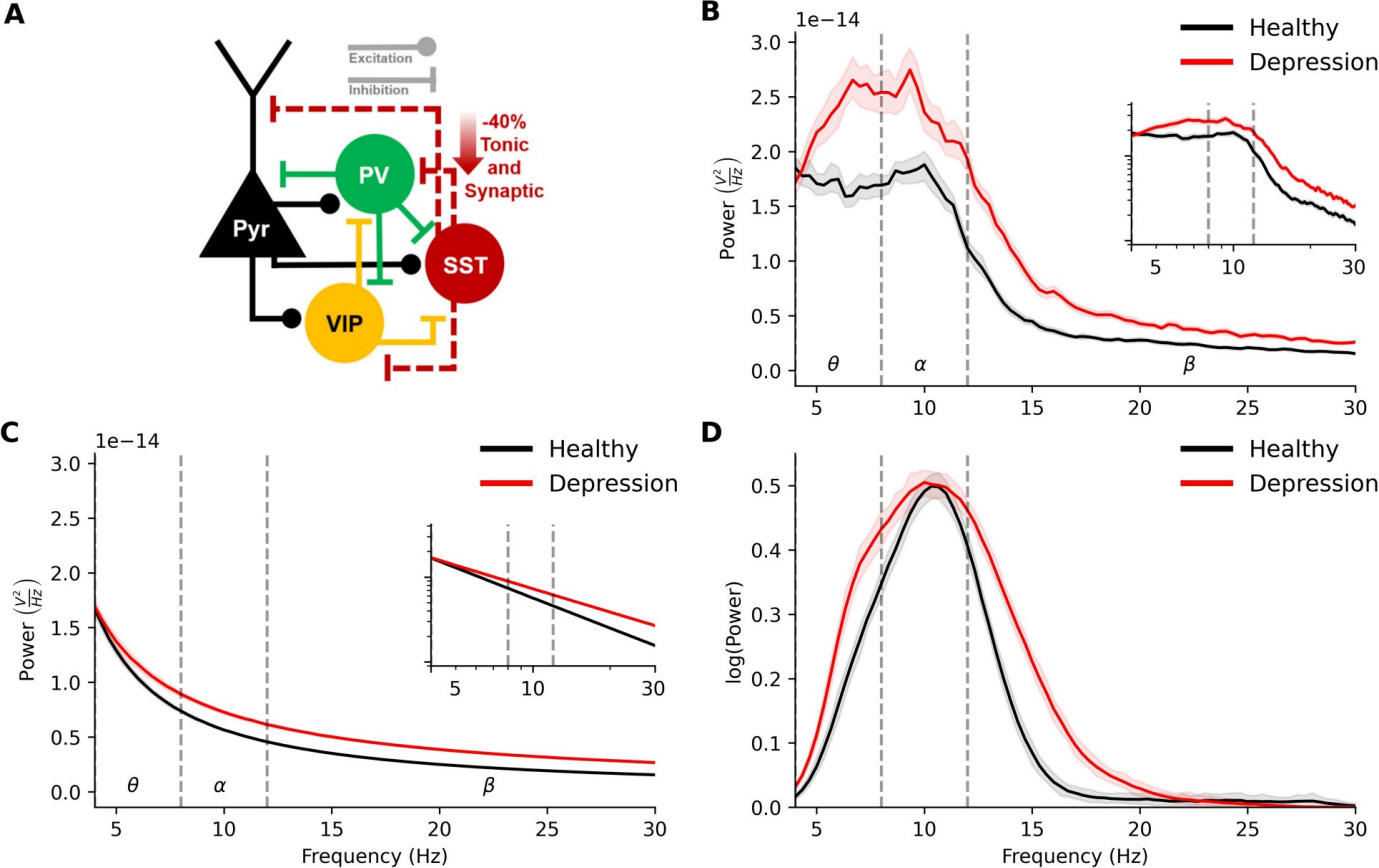
EEG signatures of reduced SST interneuron inhibition in depression microcircuit models. **(a)** Schematic connectivity diagram highlighting the key connections between different neuron types in the microcircuit. In the depression condition, dotted lines from the SST interneurons illustrate reduced synaptic and tonic inhibition onto different neuron types. **(b)** Power spectral density plot of simulated EEG from the healthy (black) and depression (red) microcircuit models (*n* = 60 randomized microcircuits per condition, bootstrapped mean and 95% confidence interval). (**c**) Fitted aperiodic component of the PSD. **(d)** Fitted periodic component of the PSD.

We compared simulated EEG in healthy versus depression microcircuits using our previous depression models, in which SST synaptic and tonic inhibition were reduced (*n* = 60 randomized microcircuits, Fig 3A). The simulated EEG from depression microcircuits exhibited a prominent peak in theta and alpha bands (4–12 Hz) similarly to the healthy microcircuits, but there was significantly increased power in these bands and in low-beta frequencies (5–16 Hz, 56% increase on average, *p* < 0.001, *d* = 1.67, Fig 3B). We decomposed EEG PSDs into aperiodic (Fig 3C) and periodic (Fig 3D) components, to compare the distinct functional components of the absolute PSDs. There was a 23% decrease in aperiodic exponent in depression compared to healthy microcircuits (healthy = 1.2 ± 0.1, depression = 0.8 ± 0.1, *p* < 0.001, *d* = 2.92), and 30% decrease in offset (healthy = 8.8*e*^−14^± 2.2*e*^−14^, depression = 6.1*e*^−14^ ± 1.6*e*^−14^, *p* < 0.001, *d* = 1.39). Together, these changes led to a 41% increase in aperiodic broadband power (5–30 Hz) in depression compared to healthy microcircuits (*p* = 0.015, *d* = 4.1). Detected peaks above the aperiodic component, which corresponded to periodic components, were clustered into canonical frequency bands based on center frequency. Depression microcircuits showed a 40% increase in peak periodic power within theta band (4–8 Hz, *p* < 0.001, *d* = 1.0), 30% increase in alpha bandwidth (8–13 Hz, *p* < 0.001, *d* = 0.7), 33% increase in peak periodic power within low-beta band (13–16 Hz, *p* = 0.002, *d* = 0.9), and 49% increase in low-beta bandwidth (*p* = 0.006, *d* = 0.8), but no significant change in alpha peak power compared to healthy microcircuits (*p* = 0.1), or center frequency for any frequency bands (*p* = 0.48). Thus, the absolute power increase involved a combination of an aperiodic broadband component, and periodic theta and low-beta components.

### EEG biomarkers of reduced SST inhibition are specific

**Fig 4:**
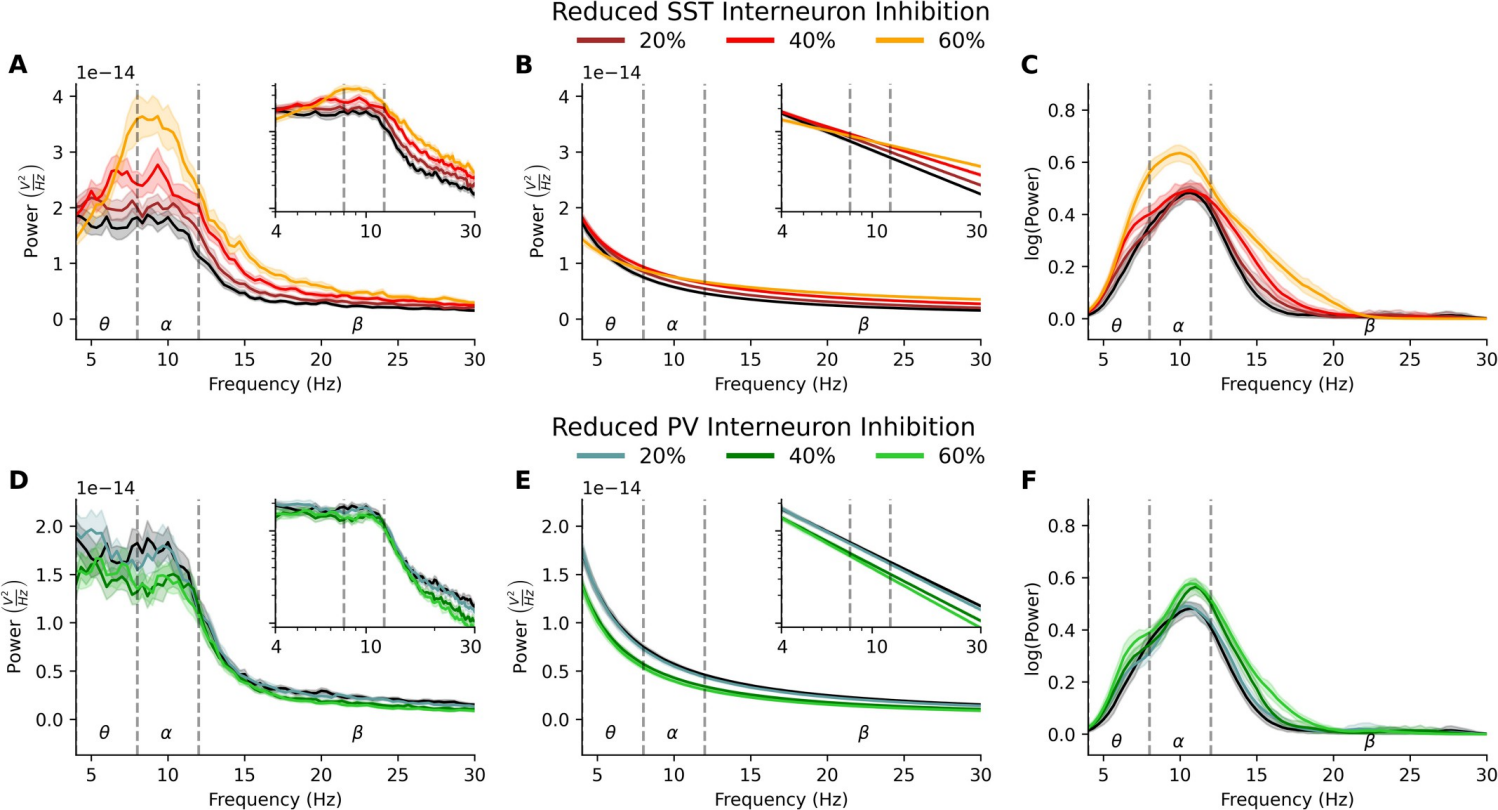
Distinct EEG signatures from reduced SST vs PV interneuron inhibition. **(a)** Power spectral density plot of simulated EEG from the healthy (black) and reduced SST interneuron inhibition (depression, red) microcircuit models, with high-low opacity indicating a 20%, 40% and 60% reduction in inhibition (*n* = 30 randomized microcircuits per condition, bootstrapped mean). **(b)** Fitted aperiodic component of the PSD. **(c)** Fitted periodic component of the PSD. **(d-f)** Same as (a-c) but for microcircuits with reduced PV interneuron inhibition.

To better establish the specificity of EEG biomarkers due to reduced SST interneuron inhibition, we simulated different levels of SST and PV interneuron inhibition reduction (20–60%) and compared their effects. Spectral profiles of reduced SST interneuron inhibition were distinct from reduced PV interneuron inhibition (Fig 4A and 4D). Absolute low-frequency power (5–16 Hz) from microcircuits with reduced PV interneuron inhibition was not significantly different from the healthy microcircuits (20–60% reduction, 8% change, *p* = 0.3), whereas PSDs from microcircuits with 20–60% reduced SST interneuron inhibition had significantly increased power (20% inhibition reduction: 24% increase, *p* = 0.03, *d =* 0.9; 60% inhibition reduction: 100% increase, *p* = 0.005, *d* = 2.4). The separability between the two types of reduced inhibition based on the raw PSD stemmed primarily from changes in decomposed aperiodic components (Fig 4B and 4E) rather than periodic components (Fig 4C and 4F). For moderate (≤ 40%) reduced inhibition, there were changes in periodic power that were unique to each cell type. Microcircuits with reduced SST interneuron inhibition showed an increase in periodic theta power compared to healthy circuits (26% increase, *p* = 0.01, *d* = 0.85), whereas microcircuits with reduced PV inhibition showed an increase in periodic alpha power (21% increase, *p* < 0.001, *d* = 1.2). However, this distinction was lost at higher (60%) levels of reduction (SST: 61% increase in theta power, *p* < 0.001, *d =* 1.48 and 34% increase in alpha power, *p* < 0.001, *d =* 1.8; PV: 31% increase in theta power, *p =* 0.002, *d =* 1.06 and 19% increase in alpha power, *p* < 0.001, *d* = 1.1). For both moderate and high levels of reduction, the estimated bandwidth of the periodic low-beta component increased only in the case of microcircuits with reduced SST interneuron inhibition (49% increase, *p* = 0.006, *d* = 0.8 and 65% increase, *p* < 0.001, *d* = 1.1), but this parameter was less reliable since it was sensitive to the fit of the primary (larger) alpha component [53]. Periodic power in microcircuits with either 20% reduced SST or PV interneuron inhibition did not significantly differ from healthy microcircuits.

In contrast, we found that the aperiodic component largely separated the two conditions (Fig 4B). Microcircuits with reduced PV interneuron inhibition showed an increase in exponent (20% inhibition reduction: 4% increase, *p* = 0.03, *d* = 0.6; 40% inhibition reduction: 7% increase, *p* < 0.001, *d* = 1.0: 60% inhibition reduction; 13% increase, *p* < 0.001, *d* = 1.8) and no significant change in offset. Contrarily, microcircuits with reduced SST interneuron inhibition showed a decrease in exponent (20% inhibition reduction: 8% decrease, *p* < 0.001, *d* = 1.3; 60% inhibition reduction: 42% decrease, *p* < 0.001, *d* = 6.6) and no significant change in offset. The aperiodic changes resulted in microcircuits with reduced PV interneuron inhibition having a decrease in broadband power (20% inhibition reduction: 6% decrease, *p* = 0.07, *d* = 1.2; 40% inhibition reduction: 27% decrease, *p* < 0.001, *d* = 5.0; 60% inhibition reduction: 36% decrease, *p* < 0.001, *d =* 6.4), whereas microcircuits with reduced SST interneuron inhibition having an increase in broadband power (20% inhibition reduction: 22% increase, *p* < 0.001, *d* = 5.2; 60% inhibition reduction: 70% increase, *p* = 0.02, *d* = 6.9). Thus, reduced SST interneuron inhibition affected the aperiodic measures of the PSD in a manner that was distinct from reduced PV interneuron inhibition.

Microcircuits with reduced PV interneuron inhibition significantly increased baseline Pyr firing rate at each step (healthy = 0.84 ± 0.03 Hz, 20% reduction: 0.86 ± 0.04 Hz, *p* = 0.008, *d* = 0.6; 40% reduction: 0.93 ± 0.04 Hz, *p* < 0.001, *d* = 1.7; 60% reduction: 1.12 ± 0.06 Hz, *p* < 0.001, *d* = 4), but to a lesser extent than reduced SST interneuron inhibition (20% SST: 1.03 ± 0.04 Hz, *p <* 0.001, *d* = 5.32; 40% SST: 1.28 ± 0.06 Hz, *p* < 0.001, *d* = 4.8; 60% SST: 1.57 ± 0.08 Hz, *p* < 0.001, *d* = 4.33). Thus, reduced SST interneuron inhibition affected baseline microcircuit activity to a greater extent than reduced PV interneuron inhibition.

**Fig 5:**
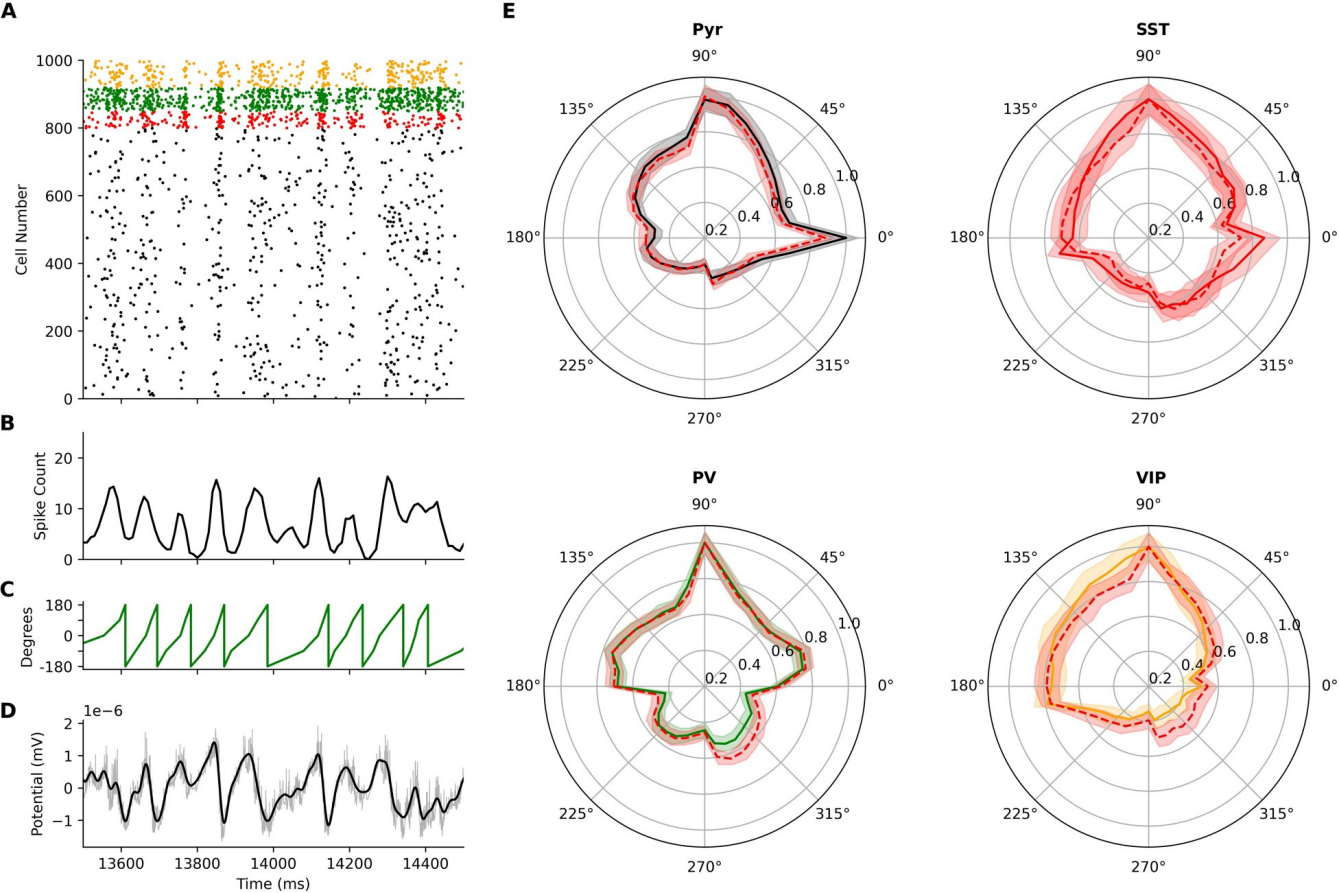
EEG phase preference of neuronal spiking in health and depression microcircuit models. **(a-d)** Temporally aligned simulated microcircuit signals used in calculating population phase-preference. **(a)** Raster plot of spiking in an example healthy microcircuit model. **(b)** Pyr population spike counts, in 10 ms bins. **(c)** Instantaneous phase of the 4–16 Hz bandpass filtered EEG signal, with trough corresponding to 0°. **(d)** Bandpass filtered EEG signal (black) and unfiltered EEG signal (gray). **(e)** Population spike counts relative to the bandpass filtered EEG from healthy (solid-line) and depression microcircuits (dotted-line). Plots show mean and standard deviation normalized by peak spike count. Neuron type colors for the healthy microcircuits are as in Fig 1.

We identified relationships between neuronal spiking and EEG spectral changes in depression microcircuit models by determining the EEG phase preference of the neuronal populations. The EEG time series were bandpass filtered from 4–16 Hz since the absolute PSDs and the periodic component of the PSDs showed highest power in these frequencies (Fig 5D). We computed the relative phase (proximity to peak, midpoint, or trough) for 4–16 Hz cycle timepoints. Spike time was converted to phase of EEG signal by temporally aligning the spike time to the instantaneous phase (Fig 5A–5C). In healthy microcircuits, spiking in all neuronal types exhibited an average preference to the phase preceding the trough of the EEG waveform (Rayleigh’s *p* < 0.001; Pyr = 66 ± 4°, SST = 62 ± 9°, PV = 79 ± 6°, VIP = 109± 5°), with a peak preference of 90° for all interneurons, and peak preferences of 0° and 90° for Pyr neurons. Depression microcircuits followed a similar phase preference (Rayleigh’s *p* < 0.001; Pyr = 72 ± 4°, SST = 67 ± 9°, PV = 72 ± 8°, VIP = 106 ± 5°), but there was a small decrease in preference selectivity, as quantified by population spike concentrations about the preferred phase for all cell types except SST interneurons (kurtosis, Pyr: 7% decrease, *p* < 0.001, *d* = 0.8; SST: 1% decrease, *p* = 0.72; PV: 21% decrease, *p* < 0.001, *d =* 1.7; VIP: 30% decrease, *p* < 0.001, *d =* 3.8).

**Fig 6:**
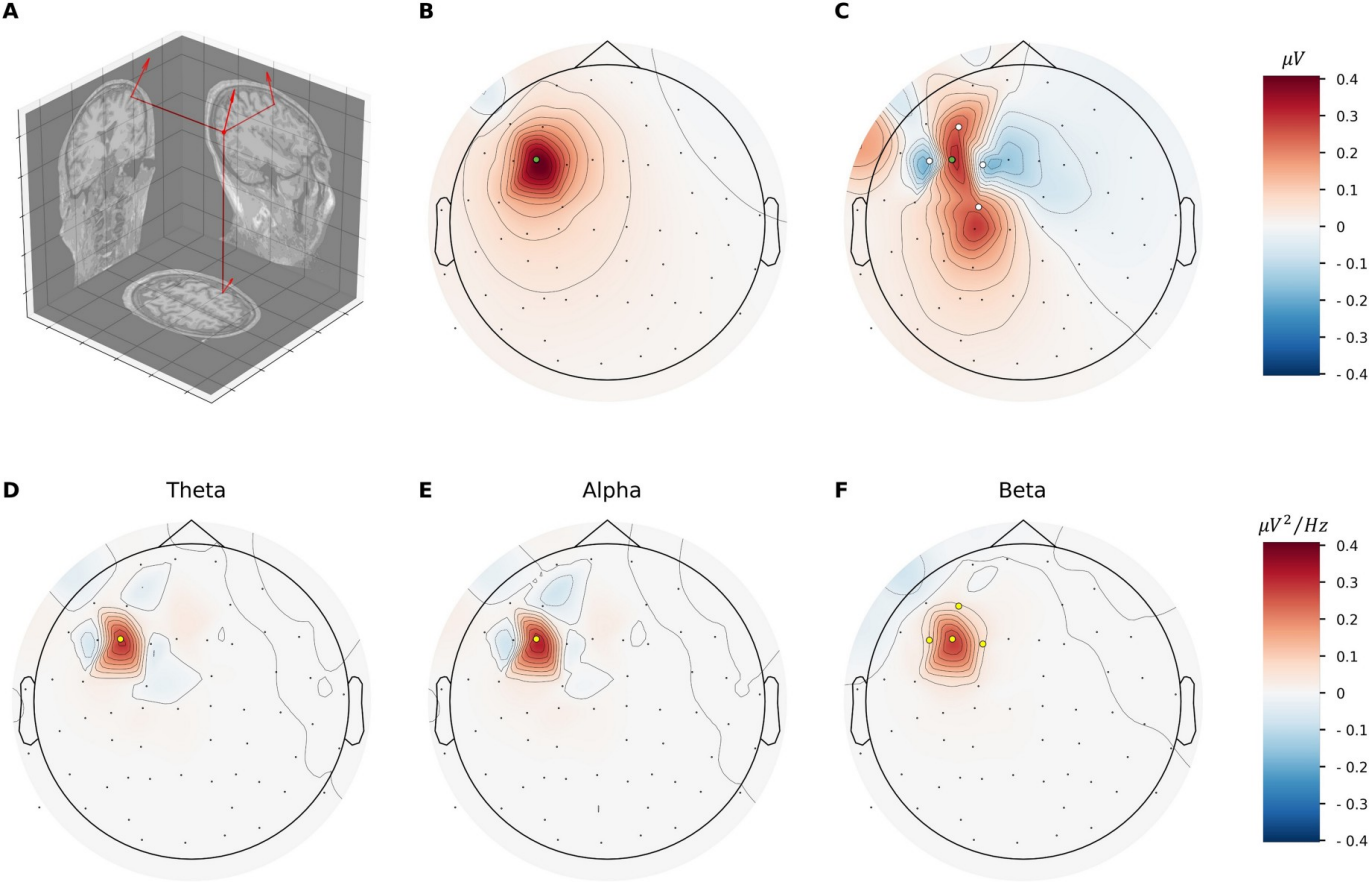
Topographic distribution of microcircuit EEG signal in a realistic head model. **(a)** Microcircuit dipole placement and orientation in the grey matter of the dlPFC in a realistic head model, perpendicular to the cortical surface. **(b)** Potential spread of the EEG signal from the microcircuit source placed under the F3 electrode (green dot) to the EEG electrodes across the head. **(c)** Example timepoint of scalp potentials from multiple sources placed under AF3, F7, F3, FC1 electrodes (white dots) surrounding the dlPFC source. Color bar applies to panels b–c. **(d)** Distribution of the difference in spectral power from depression versus healthy simulated multi-source signals for theta band (4–8 Hz). Color bar applies to panels d–f, representing difference in power with a scaling factor of 1e^-9^. **(e)** Same as (d) but for alpha band (8–12 Hz). **(f)** Same as (d) but for low-beta band (12–16 Hz).

Lastly, we examined the spatial distribution of scalp-measured EEG signals generated by the microcircuit simulations using a realistic head model. Picking an example region that is also relevant to depression, we placed the simulated microcircuit dipoles into L2/3 of the dorsolateral PFC (dlPFC), at a location underneath the 10–20 system electrode F3 (Fig 6A), and used a forward solution based on a boundary-element model with realistic head geometry and conductivity to compute simulated EEG signals. The resulting time series from this source were obtained over all EEG sensor locations (Fig 6B). We then placed simulated healthy microcircuit dipoles underneath electrodes surrounding the dlPFC region of interest (AF3, F5, F1, FC1) to provide a more realistic environment to study the potential and power differences in health and depression. Differences in theta, alpha and beta power in depression versus healthy microcircuits showed a nonuniform decay over the scalp (Fig 6D–6F). A significant difference in theta and alpha power was seen at F3 and a significant increase in beta power was seen in F3 and the neighbouring F5, F1, and FC1 electrodes (*p* < 0.001 for all). Thus, the EEG biomarkers corresponding to microcircuit changes in depression were mostly localized spatially.

## Discussion

In this study, we identified the signatures of reduced cortical inhibition in depression on EEG signals of resting-state activity using detailed models of human cortical microcircuits. Depression microcircuits with reduced SST interneuron inhibition showed an increase in absolute theta, alpha and low beta power, which involved a broadband increase together with a periodic increase in theta and low-beta activity. These EEG signatures of reduced SST interneuron inhibition were distinct from those corresponding to reduced PV interneuron inhibition, and thus provide a measure of estimating the level of cell-specific inhibition from EEG. These signatures may thus be used as biomarkers to stratify depression subtypes corresponding to altered inhibition, which are particularly associated with treatment-resistant depression [54]. By simulating both spiking and EEG in detailed microcircuits, we showed that neuronal spiking had a marked preference for the phase preceding the trough of 4–16 Hz frequencies in both conditions. Our results provide mechanistic links between reduced SST interneuron inhibition and candidate biomarkers in EEG signals. These findings have multiple clinical applications, such as improving patient stratification and monitoring the effects of therapeutic compounds targeting SST interneuron inhibition.

We examined changes in EEG PSD in terms of traditionally reported absolute power within canonical frequency bands [55] and in terms of more recent measures of decomposed aperiodic and periodic components using FOOOF [53]. Increased absolute power of theta, alpha, and low beta bands in our simulated depression microcircuits is in agreement with several previous depression studies, and was shown to be correlated with diagnosis, severity, and treatment response [7,54,56–60]. Importantly, systematic reviews across psychiatric disorders showed that these band changes are most consistent in depression and thus particular to this disorder [57]. Increased frontal theta power is predictive of second line treatment response targeting cortical inhibition in treatment-resistant depression [54,61–64]. Furthermore, depression patients who are treatment resistant show especially high theta power compared to treatment responders [54,56,58]. Together with the several points mentioned above, our results provide support for reduced SST interneuron inhibition as a mechanistic subtype of depression which is associated with treatment-resistant depression, and show that it may be detectible by a profile of increased power across the low frequency bands [6,57,59,65,66]. Increased periodic theta and low beta power, but not alpha, may explain the smaller change in relative alpha power seen previously and lend more functional significance to theta and low beta frequencies [67–70]. Future studies relating our simulated EEG biomarkers to experimental EEG and depression features will further establish the link to disease stage, subtype and severity [8,29,71,72].

Increased aperiodic broadband power and decreased aperiodic slope in our depression microcircuits are consistent with the effects of increased baseline activity and excitation-inhibition ratio, respectively [53,73,74]. Increased excitatory activity, similar phase preference but decreased selectivity in depression microcircuit models was associated with an increase in periodic activity around alpha and a broadening of alpha bandwidth. Thus, healthy and depression microcircuits had overall similar oscillatory dynamics, but spiking in the depression microcircuit was somewhat less specific, which is in agreement with leading hypothesis of increased baseline noise in spiking due to reduced inhibition [29]. In summary, our results show that altered cell-specific inhibition in microscale brain circuits in depression is reflected in key features of the EEG spectral density, which can be used to better stratify depression subtype and severity.

Reduced SST and PV circuits both increased baseline activity but to a different degree, and had distinct EEG signatures due to their specific spatial innervation on Pyr neurons [48,73,75] and thus effects on dipole moment generation. The large effect of reduced SST interneuron inhibition on the baseline firing and EEG is supported by the principal role of SST interneurons in modulating baseline cortical activity via facilitating apical dendritic synapses and lateral disynaptic inhibition [29,30,32,34,38]. Changes in SST interneuron inhibition onto apical dendrites of Pyr neuron would therefore have a strong effect on the EEG, since dipole moments increase with distance travelled by axial currents and summate both spatially and temporally [75,76]. In contrast, reduced PV interneuron inhibition had a smaller effect on baseline activity and EEG likely due to the dense PV→PV inhibitory connectivity and non-facilitating synaptic input from Pyr neurons [77], which dampened the effect of reduced inhibition, and also due to their synaptic innervation of the basal dendrites that would have a smaller effect on dipoles recorded by EEG [75].

Reduced PV interneuron inhibition had opposite broadband effects on the PSD from SST interneuron inhibition, since decreased inhibitory currents at basal dendrites would negate apical sources and decrease the amplitude of EEG activity [75]. The decreased broadband power due to reduced PV inhibition was counteracted by increased periodic power as a result of more rhythmic spike activity, leading to no significant change in absolute PSDs. Thus, by decomposing the PSD into its constituents, we further differentiated the absolute EEG signatures of reduced SST from reduced PV interneuron inhibition, highlighting this method’s utility in providing EEG correlates of altered cell-type microcircuitry. The specificity of EEG biomarkers of reduced SST inhibition conditions indicates that the biomarkers can be used to estimate the level of neuron-specific inhibition noninvasively from patient EEG and thus improve patient stratification. These results provide an important validation for our model microcircuits ability to capture a wide range of modulatory effects of distinct inhibitory populations on microcircuit oscillations.

Our biophysical models of human cortical microcircuits reproduce the two most ubiquitous features of human resting state EEG: a prominent low-frequency (4–12 Hz) peak in power and 1/f aperiodic trend [78,79]. In experimental EEG recordings, this low-frequency peak is accentuated when subjects are relaxed with their eyes closed and has the highest test-retest reliability of all spectral features [80]. While recurrent rhythmic activity generated within cortico-thalamic loops is believed to contribute strongly to scalp-measured EEG alpha, cortico-cortical interactions have also been shown to generate alpha activity in top-down processing [81]. Further, it has been shown that cortical Pyr neurons significantly contribute to and sustain theta and alpha-phasic firing due to intrinsic properties and recurrent activity. The key features of human EEG reproduced by our microcircuit models were not explicitly constrained for, but rather emerged from the interaction of human neuronal electrical properties, synaptic connectivity, and baseline firing rates. This provides a validation of the models and indicates that they contain circuit motifs responsible for generating realistic microcircuit activity, and it can therefore be viewed as a useful canonical cortical microcircuit model irrespective of layer considerations, in line with previous studies [49,81–83]. It will be of interest in future studies to use the models for further elucidating the cellular mechanisms of oscillatory generation, especially in brain states other than rest and during cortical processing, which involve other frequency bands. In addition, our models captured other periodic features seen in experimental literature, such as alpha bandwidth [84]. However, these features are especially sensitive to recording quality and modelling parameters [85]. As our models capture the key spectral EEG features seen *in vivo*, the simulated EEG signal magnitude can also be related to that of the recorded EEG by scaling the signal by 10,000–100,000, to account for the difference between the number of neurons in our models (1000) compared to the millions of neurons that generate the recorded EEG at a given electrode [37,45]. Alternatively, one may use relative frequency power from our microcircuit PSD to compare to previous clinical findings [55].

Linking altered mechanisms at the microcircuit scale to EEG biomarkers is currently impossible to establish experimentally in humans, thereby meriting the use of data-driven detailed computational studies. Our previous work discusses modelling considerations in detail [33]. Briefly, we have estimated reduction of SST interneuron synaptic and tonic inhibition conductance from gene expression data [16] as supported by the co-localization and co-release of GABA and SST pre- and post-synaptic [19], and by rodent animal models of depression [14,86,87]. We have also simulated multiple levels of reduced inhibition to better quantify the relationship between levels of reduced inhibition and resulting EEG changes. We used a simplified model of tonic inhibition as a steady-state current due to its long timescale [88].

We simulated human EEG using available detailed models of human cortical L2/3 microcircuits to serve as canonical cortical microcircuit models. These layers are closest to the electrode and thus are important contributors to the EEG signal, along with L5/6 [89]. Previous computational studies have shown that interactions between superficial and deep layers are important contributors to electrophysiological signals, especially in other contexts such as event-related potentials [44]. As new human cellular data becomes available, future models that also include deeper layers (4–6) may bolster narrow-band oscillatory activity to EEG, such as beta activity [81,90]. Relatedly, including deeper layers will provide insight on laminar interactions mediated by SST interneurons and their contributions to EEG in health and depression [45,91]. Future studies may further refine our biomarkers by modeling other mechanisms of depression, such as synapse loss [92]. Through coupling the biophysical simulations with a forward solution using a realistic head model we showed that EEG signals would be fairly localized to the source and neighboring electrodes. This computational approach can serve to further study source localization in interpreting EEG recordings, improve inverse solutions [93], and give greater physiological interpretability to statistical decomposition techniques such as independent component analysis [94] or s/eLORETA [95]. While we modeled and studied a single cortical region at the microcircuit scale, future studies could simulate several distinct microcircuits to examine how altered circuit mechanisms affect multi-regional interactions in depression [29].

Using detailed multi-scale models of human cortical microcircuits, we were able to characterize the EEG and spike correlates of reduced SST interneuron inhibition in depression. Previous modeling studies have shown that reduced SST interneuron inhibition in depression impairs stimulus processing due to increased baseline activity and noise [33]. Our models can serve to identify corresponding biomarkers in task EEG. The computational models we have developed also provide a powerful tool to identify the EEG biomarkers of novel therapeutic compounds and treatments for depression [21,96] via *in silico* simulations to improve treatment monitoring. Finally, our models and methodology may further serve to identify EEG biomarkers of altered cellular and circuit mechanisms in other neurological diseases, such as epilepsy and schizophrenia.

## Methods

### Human cortical microcircuit models

We used our previous models of human cortical L2/3 microcircuits [33], consisting of 1000 neurons distributed in a 500x500x950μm^3^ volume (250 to 1200μm below pia [97]). All model assumptions, parameter choices, and simulation methodology followed our previous work [33]. Briefly, the model microcircuits included the four key neuron types in cortical L2/3: Pyramidal (Pyr), Somatostatin-expressing (SST), Parvalbumin-expressing (PV), and Vasoactive Intestinal Peptide-expressing (VIP) neurons. The proportions of the neuron types were: 80% Pyr, 5% SST, 7% PV, and 8% VIP in accordance with relative L2/3 neuron densities [98,99] and RNA-seq data [100,101]. The models were simulated using *NEURON* version 7.8.0.119 [102] and *LFPy* version 2.0.2 [103]. The multicompartmental conductance-based models of human neurons included human neuron morphologies from the Alan Brain Atlas [100,104], and reproduced firing properties and dendritic sag measured in human neurons. The models also reproduced synaptic properties (post-synaptic potential amplitudes and short-term dynamics) as measured in human neuron pair-recordings between Pyr neurons, PV and SST interneurons [31,46,105,106]. Importantly, the models captured the key disynaptic loop motif in humans by reproducing Pyr ➔ SST ➔ Pyr synaptic properties, whereby a train of spikes in one Pyr neuron triggered a spike in an SST interneuron and the experimental amplitude of inhibitory postsynaptic potentials in neighboring Pyr neurons [31]. The models included previous AMPA/NMDA excitatory and GABA_A_ inhibitory synapse mechanisms (*τ*_*rise*,*NMDA*_ = 2 ms; *τ*_*decay*,*NMDA*_ = 65 ms; *τ*_*rise*,*AMPA*_ = 0.3 ms; *τ*_*decay*,*AMPA*_ = 3 ms; *τ*_*rise*,*GABA*_ = 1 ms; *τ*_*decay*,*GABA*_ = 10 ms; reversal potentials *E*_*exc*_ = 0 mV and *E*_*inh*_ = -80 mV) [49,107,108]. Connection probabilities for Pyr ➔ Pyr connection were set to 15% according to human literature [46], with others according to rodent literature [100].

### Resting-state activity simulations

We simulated eyes-closed resting-state by injecting the microcircuit models with background excitatory input representing cortical and thalamic drive, as used previously [33]. The background input was generated by random Orstein-Uhlenbeck (OU) point processes [109] placed on all neurons at the halfway point of each dendritic arbor, and 5 additional OU processes placed along the apical trunk of Pyr neurons in equal intervals, from 10% to 90% apical dendritic length. This enabled the circuit to generate recurrent activity with neuronal firing rates as measured *in vivo* [32,33,110,111]. For both healthy and depression microcircuit models (see below) we simulated 60 randomized microcircuits, for 28 seconds each.

### Microcircuit models with reduced SST interneuron inhibition (depression microcircuits)

We used previous models of depression microcircuits [33], with 40% reduced synaptic and tonic inhibition from SST interneurons onto all other neurons, in accordance with gene expression studies in SST interneurons in post-mortem brains from depression patients [16]. For Pyr neurons, we decreased apical tonic inhibition by 40%. For each interneuron, we decreased the relative SST contribution to tonic inhibition by 40%. To compare reduced SST from reduced PV interneuron inhibition, we then decreased synaptic and tonic inhibition from SST interneurons by 20%, 40%, and 60%.

### Microcircuits with reduced PV interneuron inhibition

Reduced PV interneuron inhibition was modeled by iteratively reducing synaptic and tonic inhibition from PV interneurons onto all neurons by 20%, 40%, and 60%.

### Simulated microcircuit EEG

We simulated layer-averaged dipole moments together with neuronal activity using *LFPy* [48]. The full methodology and equations for calculating dipole moments are described in Linden at al 2010 [112] as well as Hagen et al 2018 section 2.3.1 and 2.5.10 [48]. Briefly, dipole moments for each cell were computed from transmembrane currents:

$$p(t)=\sum_{n=1}^{n^{seg}}r_{n}I_{n}^{m}\;(t)$$

where $I_{n}^{m}$ is the transmembrane current at time *t* from compartment *n* at position *r*_*n*_. This was done for each x, y, z component of the dipole moment, for each timestep, at each segment.

Corresponding EEG timeseries were generated from dipole moment timeseries using a four-sphere volume conductor model that assumes homogeneous, isotropic, and linear (frequency independent) conductivity for each medium. A four-sphere head model accurately captures EEG for sources directly under the scalp electrode [48,78,113]. The radii of the four spheres representing the brain (grey and white matter), cerebrospinal fluid, skull, and scalp were 79 *mm*, 80 *mm*, 85 *mm*, and 90 *mm*, respectively. The conductivity for each sphere was 0.47 S/m, 1.71 S/m, 0.02 S/m, and 0.41 S/m, respectively [113,114]. A fixed dipole origin was placed at the midpoint of L2/3 (-725 *μm*) oriented normal to the surface of the scalp. The extracellular potential measured from an EEG electrode on the skin placed directly above the circuit was calculated using the following equations:

$$\phi^{1}(r,\theta )=\frac{p}{4\pi \sigma_{1}r_{z}^{2}}\sum_{n=1}^{\infty }[A_{n}^{1}{\left(\frac{r}{r_{1}}\right)}^{n}+{\left(\frac{r_{z}}{r}\right)}^{n+1}]nP_{n}(cos\theta ),when\;r_{z}<r\leq r_{1}$$

$$\phi^{s}(r,\theta )=\frac{p}{4\pi \sigma_{1}r_{z}^{2}}\sum_{n=1}^{\infty }[A_{n}^{s}{\left(\frac{r}{r_{s}}\right)}^{n}+B_{n}^{s}{\left(\frac{r_{s}}{r}\right)}^{n+1}]nP_{n}(cos\theta ),when\;r_{s-1}<r\leq r_{s}$$

where *r* is the distance of the electrode from the center of the brain sphere, and *θ* is the angular separation between the dipole and the electrode. The first equation describes the potential in the innermost sphere (brain tissue, s = 1), *ϕ*^1^(*r*, *θ*), and the second describes the potential in the other 3 concentric shells (cerebrospinal fluid, skull, and scalp, s = 2,3,4), *ϕ*^*s*^(*r*, *θ*). The potential was measured at the extracellular electrode, *ϕ*^*s*^(*r*, *θ*), using a current dipole moment of magnitude *p* at a radial location *r*_*z*_. Since shell conductivity, *σ*_*s*_, and radius, *r*_*s*_, affect the signal propagation, $A_{n}^{s}$ and $B_{n}^{s}$ are introduced as scaling coefficients to account for these effects in each shell. *P*_*n*_(*cosθ*) is the *n* Legendre Polynomial. Full methodology for computing $A_{n}^{s}$ and $B_{n}^{s}$ for relevant shells is outlined in equations 7–16 in Næss et al 2017 [113]. These equations for the extracellular potential of a radial dipole accounted for the key component of the simulated EEG signal from the oriented dipole, and were used together with similar equations for tangential dipoles as outlined in equations 17 and 18 of Næss et al 2017 [113]. EEG power spectral density (PSD) and spectrograms were calculated using Welch’s method [115], with a 3s second Hanning window for PSD, and 0.5s Hanning window for spectrograms. Both analyses used 30% window overlap.

### Quantifying high power events

EEG timeseries were bandpass filtered into their canonical frequency bands. The envelope for each band was calculated using the real component of the Hilbert transform from SciPy version 1.4.1 [116]. High power events were defined as periods between where the envelope height passed a threshold of 1 standard deviation above the mean envelope height, and which lasted more than 100 ms.

### EEG periodic and aperiodic components

We decomposed EEG PSDs into periodic and aperiodic (broadband) components using the standard method described in the FOOOF package, version 1.0.0 [53]. The aperiodic component of the PSD was a 1/f function, defined by a vertical offset and exponent parameter. After removing the aperiodic component from the PSDs, we derived from the flattened spectrum the periodic components (representing putative oscillations) using gaussians, defined by center frequency (mean), bandwidth (variance), and power (height). We used the following settings for the algorithm: peak width limits = [2, 6]; max number of peaks = 3; minimum peak height = 0; peak threshold = 2; and aperiodic mode = ’fixed’. Power spectra were fit across the frequency range 3–30 Hz with a resolution of 0.33 Hz. The aperiodic fit was refined for a better fit using the peak-removed spectrum.

### Population spiking phase preference

We calculated the instantaneous phase of EEG using the ByCycle python library version 1.0.0 [117]. Briefly, EEG time series were bandpass filtered from 4–30 Hz to isolate the oscillatory component of the EEG wave while omitting higher frequencies. Peaks and troughs between narrow 4–16 Hz zero-crossings were identified. Local rise and decay midpoints were then identified as the midpoint between the EEG cycle’s local maxima and minima. 0° was defined as the *cos* EEG cycle start (peak) and trough corresponding to 180°. For each of the four neuron populations (Pyr, SST, PV, VIP), spike times were aggregated into a population spike vector and converted to corresponding phase of EEG. Spike phases were binned into 10° bins and the counts were summated over all circuits in each condition. To compare spike preference between conditions, bin counts were normalized by the maximal count.

### Simulated EEG from an anatomically detailed head model

Whereas a four-sphere head model is suitable for simulating EEG from microcircuit sources directly under the scalp electrode, head shape affects EEG signal spread and thus the contribution of signals from more distant sources to the electrode [75]. To study the EEG signal decay over the scalp, we generated EEG using an anatomically detailed head model. The x-y-z components of microcircuit dipole moments generated from *LFPy* and used in the four-sphere head model were imported into *MNE* version 0.24.0 [118] and downsampled to a 100 Hz sampling rate. The dipole was placed underneath the F3 electrode, corresponding to the dlPFC [119] normal to the gyrus surface. We chose to examine this region because it has been implicated in depression [120], and shows a possible decrease in SST inhibition [17]. We solved the forward solution using a three-layer boundary element model, as implemented in LFPy, corresponding to the inner skull, outer skull, and scalp with conductivities of 0.3 S/m, 0.006 S/m and 0.3 S/m, respectively. The resulting potential was calculated for a standard 64-channel 10–20 EEG system. Sensor PSDs were calculated using Welch’s method, as in the four-sphere head model. To examine differences between healthy and depression microcircuits in a more realistic multi-regional context, 4 random healthy microcircuit dipole moment timeseries were placed under electrodes surrounding the dlPFC (F5, F1, AF3, FC1) and resulting potentials over the 64 channel EEG system were collected (n = 60 randomized microcircuits for each of the source electrodes). In the depression condition, we placed a depression microcircuit dipole moment timeseries under F3 and random healthy microcircuit timeseries under the surrounding electrodes. Timeseries for surrounding regions were randomly chosen from a pool of 60 time-series with the condition that they were not duplicated within a single simulation. We tested sensor-wise significance of depression vs healthy by comparing to the healthy band power and noise, as estimated by dlPFC source power and the additional noise level provided by the surrounding random healthy circuit signals.

### Statistical tests

We used two-sample t-test to determine statistical significance where appropriate. We calculated Cohen’s *d* to show effect size. We used Raileigh’s test of non-uniformity to determine if populations had a non-uniform phase preference, and kurtosis to quantify variance around the preferred mean angle.
